# Editorial: Defect chemistry in electrocatalysis

**DOI:** 10.3389/fchem.2022.1118783

**Published:** 2022-12-16

**Authors:** Dafeng Yan, Longlu Wang, Feng Zeng, Huawei Huang

**Affiliations:** ^1^ College of Chemistry and Chemical Engineering, Hubei University, Wuhan, China; ^2^ College of Flexible Electronics (Future Technology), Nanjing University of Posts and Telecommunications (NJUPT), Nanjing, China; ^3^ State Key Laboratory of Materials-Oriented Chemical Engineering, College of Chemical Engineering, Nanjing Tech University, Nanjing, Jiangsu, China; ^4^ KAUST Catalysis Center (KCC), King Abdullah University of Science and Technology (KAUST), Thuwal, Saudi Arabia

**Keywords:** electrocatalysis, defect engineering, defect chemistry, electrocatalyst, energy storage and conversion

With the fast development of modern society, environmental pollution and energy crisis have been drawing broad attention. It is highly desirable to develop renewable and clean energy conversion and storage technologies to overcome these issues (Yan et al., 2022; Zhang et al., 2016). Electrochemical energy storage and conversion devices show great potential for their intrinsic advantages, such as low cost, environment-friendly, and renewable (Chen and Shi, 2022). Electrocatalysis plays a vital role in electrochemical energy storage and conversion devices. Since the performances of these devices are greatly limited by the electrochemical reactions, it is very necessary to design highly efficient electrocatalysts to promote these reactions (Yan et al., 2022a). As the electrocatalytic reactions occur on the surface of the catalysts, the surface electronic structure of electrocatalysts largely determines their performance (Xie et al., 2021).

Among various strategies to modulate the electronic structure of electrocatalysts, defect engineering has recently become a very hot topic and draws much attention to the application of electrocatalytic fields (Yan et al., 2022b; Yan et al., 2017; Yan et al., 2019). In recent years, many researches related to defect chemistry in electrocatalysis were published and cited, which indicated that this topic is very hot and has drawn much attention (Li et al., 2022c). Though many researchers have paid their attention to defect chemistry in electrocatalysis, it is still in the initial period and there is great space for exploration in the aspects of synthetic method, structure characterization and dynamic evolution during different electrocatalytic reactions.

Herein, we collected five valuable contributions focusing on the defect chemistry of different materials and different electrocatalytic reactions. This special Research Topic includes four reviews and one original research. Defect engineering on carbon-based and metal-based single-atom electrocatalysts was highlighted by (Li et al., 2022b) along with the discussions of their characterization. Defective Noble metal and non-noble metal catalysts for electrocatalytic oxygen reduction reaction were discussed and summarized by (Mao et al., 2022) Based on experimental results and theoretical calculations, they clarified the structure-activity relationships between defect engineering and catalytic performance. Oxygen vacancies engineering in electrocatalysts for nitrogen reduction reaction was summarized by (Zhu et al., 2022) They focused on the methods to generate oxygen vacancies and their effects on electrocatalytic nitrogen reduction reaction. (Li et al., 2022a) briefly summarized recent research progress in defect engineering of electrocatalysts for electrochemical CO_2_ reduction. They summarized various strategies for adjusting and modifying the surface defects of catalysts, including intrinsic defects, heteroatom doping, single-metal-atom sites, vacancies, grain boundaries and lattice defects. (Xiao et al., 2022) successfully introduced the doughty-electronegative heteroatom defect to MoS_2_ through a large-scale and simple ball milling strategy, which can greatly enhance the hydrogen evolution reaction performance.

As guest editors, we would like to thank all the authors for their valuable contributions to this Research Topic and all the reviewers for their important and thoughtful comments. We hope that this Research Topic will provide useful insights for the development of and help readers understand more about the effects of defect chemistry on different electrocatalytic reactions.
